# The Associations of Dietary Iron Intake and the Transferrin Receptor (*TFRC*) rs9846149 Polymorphism with the Risk of Gastric Cancer: A Case–Control Study Conducted in Korea

**DOI:** 10.3390/nu13082600

**Published:** 2021-07-28

**Authors:** Tao Thi Tran, Madhawa Gunathilake, Jeonghee Lee, Il Ju Choi, Young-Il Kim, Jeongseon Kim

**Affiliations:** 1 Department of Cancer Control and Population Health, Graduate School of Cancer Science and Policy, Goyang-si 10408, Gyeonggi-do, Korea; 2006101@ncc.re.kr; 2 Department of Cancer Biomedical Science, Graduate School of Cancer Science and Policy, Goyang-si 10408, Gyeonggi-do, Korea; 75792@ncc.re.kr (M.G.); jeonghee@ncc.re.kr (J.L.); 3 Center for Gastric Cancer, National Cancer Center Hospital, National Cancer Center, Goyang-si 10408, Gyeonggi-do, Korea; cij1224@ncc.re.kr (I.J.C.); 11996@ncc.re.kr (Y.-I.K.)

**Keywords:** iron, nonheme iron, gastric cancer, case–control study, rs9846149

## Abstract

Background: A positive association between a high iron intake and colorectal cancer has been identified; however, the effect of dietary iron on gastric cancer (GC) remains unclear. Here, we investigate whether dietary iron is related to GC risk and whether the transferrin receptor (*TFRC*) rs9846149 polymorphism modifies this association. Methods: A case–control study was designed to assess this association among 374 GC patients and 754 healthy controls. A self-administered questionnaire was used to collect information on demographics, medical history and lifestyle. Dietary iron intake was assessed using a semi-quantitative food frequency questionnaire. *TFRC* rs9846149 was genetically analyzed using the Affymetrix Axiom Exom 319 Array platform. Results: A higher total dietary iron was significantly associated with decreased GC risk [OR = 0.65 (0.45–0.94), *p* for trend = 0.018]. A similar association was observed with nonheme iron [OR = 0.64 (0.44–0.92), *p* for trend = 0.018]. Individuals with a major allele of *TFRC* rs9846149 (CC/GC) and higher intake of total iron had a significantly lower GC risk than those with a lower intake [OR = 0.60 (0.41–0.88), *p* interaction = 0.035]. Conclusion: Our findings show the protective effects of total dietary iron, especially nonheme iron, against GC risk, and this association can be modified by *TFRC* rs9846149.

## 1. Introduction

According to GLOBOCAN, gastric cancer (GC) was one of the most common cancers and one of the main causes of mortality related to cancer worldwide in 2020 [1]. In Korea, although the GC incidence has decreased steadily since 1999, it is still a common cancer [2]. The age-standardized incidence rates have been reported to be approximately 44.3/100,000 among males and 18.3/100,000 among females [2].

The trends of GC are related to recognized risk factors. Helicobacter pylori (H. pylori), smoking status and a diet with high salt and processed meat consumption have been strongly indicated as risk factors [3]. In contrast, a healthy diet supplies vital nutrients, which have been reported to be necessary for cancer prevention [4,5].

Among the vital nutrients, iron is a necessary element for organisms. Its importance has been well documented in the literature. Iron is known to play an important role in transporting oxygen and electrons and synthesizing deoxyribonucleic acid (DNA) [6]. However, the excessive consumption of iron may be a risk factor for cancer development; the role of iron as a catalytic agent in hydroxyl radical formation, in the suppression of host cell defense activity, and in boosting malignant proliferation are widely accepted as mechanisms [7,8,9]. Therefore, attention has been drawn to the specific relationship of GC with iron metabolism in recent years.

*Transferrin receptor 1 (TFR1)* is encoded by the *TFRC* gene, which is responsible for importing iron by binding transferrin (TF). Iron uptake by *TFR1* is strongly recognized as an important mechanism of iron absorption by cancer cells. Iron uptake by *TFR1* affects the proliferation, migration, invasion, and metastasis of cancer cells. The aforementioned mechanisms have been reinforced by conclusions of recent studies. In detail, high expression of *TFR1* was observed in individuals with GC as well as those with other cancers [10,11,12,13,14]. Furthermore, overexpression of the *TFRC* gene was observed in *H. pylori*-positive tissues [15]. Thus, we hypothesized that the interaction between *TFRC* and iron may play an important role in the different outcomes observed.

*H. pylori* bacteria colonize the stomach [16], and genes related to iron uptake play a vital role in its colonization. There is competition for obtaining iron between humans and the bacteria, resulting in gene expression, including *TFRC*. In an environment without free iron, *H. pylori* will acquire iron from proteins, and *TFRC* will be used as an element for attachment and pathogenesis [15]. Single nucleotide polymorphisms (SNPs), which are the most common type of genetic mutation, may influence susceptibility to GC [17]. The effects of exposure to environmental factors may be modulated by genetic variants through the regulation of multiple biological pathways during gastric carcinogenesis [18]. rs9846149 is a common variant found in the *TFRC* gene among the Korean population. rs9846149 contains a C allele and a G allele; specifically, C is considered the wild type, and the G allele is a mutant type. Therefore, we have 3 genotypes (CC, CG, and GG) in the population. We established the hypothesis that the association of *TFRC* rs9846149 with GC may be affected by iron intake due to the aforementioned mechanisms.

To our knowledge, the interaction effect between dietary iron and the *TFRC* gene on GC risk modification has not been investigated in epidemiological studies. Moreover, despite the strong biological mechanisms, observational studies failed to obtain consistent findings regarding the relationship of dietary iron with the risk of GC [4,9,19,20,21,22]. Importantly, the majority of studies have focused on heme iron rather than nonheme iron [9,22,23,24]. Thus, this association is still open to discussion. Therefore, our study aimed to examine whether dietary iron is related to GC risk and whether the *TFRC* rs9846149 polymorphism modifies this association.

## 2. Materials and Methods

### 2.1. Study Design and Participants

We conducted this study between March 2011 and December 2014 at National Cancer Center Hospital in Korea. Patients who were diagnosed with GC no longer than 3 months before recruitment were defined as cases. Patients with chronic diseases, advanced GC or previous diagnosis of any cancer were excluded because they may have changed their eating habits. Pregnant and currently breastfeeding women were also excluded. We selected the control subjects from the pool of individuals undergoing health screening examinations at the same hospital without any diagnosis of cancer or chronic diseases, and cases were matched with controls by sex and age (±5 years); the ratio was 1:2. In total, 374 GC cases and 754 healthy controls with available data for dietary iron and genetic information were included in the analysis. We obtained informed consent from all participants. The study protocol was approved by the Institutional Review Board of the National Cancer Center (NCCNCS 11-438).

### 2.2. Dietary Measurement

Dietary intake information within the past 12 months regarding the frequency of food consumption (never or rarely, once a month, 2–3 times a month, once or twice a week, 3–4 times a week, 5–6 times a week, once a day, twice a day, and 3 times a day) and portion sizes (small, medium, and large) was obtained from all participants by using a semi-quantitative food frequency questionnaire (SQFFQ) with 106 food items. The information was collected by a trained interviewer. The assessment of the validity and reliability of the SQFFQ has been reported previously [25]. The average nutrient intake/day was calculated based on the serving of foods consumed each time, consumption frequency and the nutritional content of food items. CAN-Pro 4.0 (ComputerAided Nutritional Analysis Program, The Korean Nutrition Society, Seoul, Korea) was used for this calculation. The sum of the amount of iron obtained from all of the food consumed in the day was considered the dietary iron intake (mg/d). Moreover, subjects were required to complete a self-administered questionnaire regarding demographic and lifestyle information.

### 2.3. Genotype Measurement

The genotyping and quality control processes have been described in previous studies [26,27,28]. Extraction of genomic DNA was performed using peripheral blood leukocytes. The Affymetrix Axiom Exom 319 Array (Affymetrix Inc., Santa Clara, CA, USA) platform, including 318,983 variants, was used for genotyping. Genetic markers with deviation from Hardy–Weinberg equilibrium *p*-values < 1 × 10^–6^, a minor allele frequency (MAF) < 0.01, and a low call rate (<98%) were discarded. The Asian population (*n* = 504) in the 1000 Genome haplotypes phase III integrated variant set release GRch37/hg19 (https://www.1000genomes.org/, accessed on 13 June 2016) was used as a reference panel for genotype imputation. Phasing and SNPs imputation were performed; SHAPIT (v2.r837) was used for the former, and IMPUTE2 (2.3.2) was used for the latter. The application of quality control criteria was considered after filtering for an INFO score over 0.6. Finally, we selected the genetic polymorphism rs9846149.

### 2.4. Statistical Analyses

Iron intake was adjusted for total energy intake by using a residual method [29]. The distribution in the control group served as a criterion for categorizing dietary iron into tertiles. We used the chi-square test for categorical variables and the *t*-test for continuous variables, including iron consumption, to analyze the differences in general characteristics between cases and controls. We performed a test for trends by using the median value of each tertile category as a continuous variable to determine the dose–response relationships of dietary iron with GC risk. We used logistic regression models with odds ratios (ORs) and 95% confidence intervals (CIs) to explore the association (Model I: crude model; Model II: adjusted for age, first-degree family history of GC [FDFHGC], BMI, smoking status, alcohol consumption, education, occupation, and monthly income; and Model III: additionally adjusted for *H. pylori* infection. In the overall subjects, models II and III were additionally adjusted for gender). The lowest tertile group was considered the reference group. Missing values for each variable were kept as a category. We used recessive and dominant models to analyze genetic associations. Tertiles were used for analysis of the effect of the interaction between dietary iron and genetic polymorphisms. The likelihood ratio test between the models with and without the interaction term (Iron*SNPs) was used to analyze the interaction. SAS software (version 9.4, SAS Institute, Cary, NC, USA) was used for all statistical analyses, and a two-sided *p*-value less than 0.05 was considered significant.

## 3. Results

### 3.1. General Information of Participants

Table 1 represents the information of the subjects according to the selected variables. Out of the 354 cases and 754 controls, a higher percentage of *H. pylori*-positive individuals was found in the GC group (*p* < 0.001). The pattern was repeated for FDFHGC (*p* < 0.001) and current smoking status (*p* < 0.001). Lower levels of regular exercise, education, occupation and monthly income (*p* < 0.001) were also observed in the GC group (*p* < 0.001). Compared with males in the control group, males in the case group exhibited a higher rate of positive *H. pylori,* FDFHGC, current smoking, nonregular exercise, low education level, low income level and high occupational level. Similarly, the proportion of these characteristics in female cases was significantly higher than in healthy females.

Table 2 shows the difference in total energy and iron intake between the two groups. Compared with healthy controls, GC patients exhibited significantly higher total energy intake (*p* < 0.001) and significantly lower total dietary iron intake (*p* = 0.005). Moreover, nonheme iron intake was significantly lower in GC cases than in the controls (*p* = 0.008). Compared with healthy females, females with GC consumed less total iron (*p* = 0.021) and nonheme iron (*p* = 0.047).

Table 3 represents the association of total iron intake, nonheme iron intake, and heme iron intake with risk of GC. In terms of total dietary iron, a significant inverse trend with the risk of GC was consistently found in both the crude model and fully adjusted model, and the ORs (95% CI) were 0.60 (0.44–0.82) and 0.65 (0.45–0.94), respectively (*p* for trend < 0.05). The association appeared to be restricted to nonheme iron, for which a protective effect was found (OR = 0.64, 95% CI = 0.44–0.92, *p* for trend = 0.018), but the relationship was null for heme iron.

A reduced risk was observed for female participants with a higher consumption of total iron compared with those with a lower consumption (OR = 0.50, 95% CI = 0.30–0.85, *p* for trend = 0.010), but the significant relation disappeared after additional confounders were adjusted (OR = 0.61, 95% CI = 0.33–1.13, *p* for trend = 0.116). Nonheme iron exhibited a similar result; in the fully adjusted model, an association of nonheme iron with a reduction in GC risk was not found in females (OR = 0.74, 95% CI = 0.40–1.36, *p* for trend = 0.408) (Table 3).

We then performed subgroup analyses based on *H. pylori* infection status, regular exercise, smoking status, and FDFHGC to observe the association between dietary iron intake and GC risk. In the *H. pylori*-positive group, a protective effect against GC was found for total iron (OR = 0.66, 95% CI = 0.44–0.98, *p* for trend = 0.035). However, we failed to find a significant association for nonheme iron (Supplementary material Table S1). When stratified by smoking status, a negative relationship of total iron with GC risk was observed for ever-smokers (OR = 0.51, 95% CI = 0.29–0.88, *p* for trend = 0.016). With regard to nonheme iron, the risk also tended to decrease, although a significant trend was reported only for males (OR = 0.55, 95% CI = 0.33–0.93, *p* for trend = 0.028) (Supplementary material Table S2). We assessed the protective effects of total iron and nonheme iron among those who performed regular exercise, and did not have FDFHGC. Although a lack of homogeneity was observed for males and females, a significant association emerged for males, and the corresponding values in the group of regular exercisers were 0.41 (95% CI = 0.20–0.85, *p* for trend = 0.009) for total iron and 0.29 (95% CI = 0.14–0.60, *p* for trend = 0.002) for nonheme iron (Supplementary Materials Tables S3 and S4).

### 3.2. The Associations of the TFRC rs9846149 Polymorphism with Gastric Cancer Risk

A majority of the studied population had a major allele of *TFRC* rs9846149 (CC/GC) (95% for controls and 95.7% for cases). Only 5% of participants had GG homozygotes. With regard to the recessive model, no significant association of the *TFRC* rs9846149 polymorphism with GC risk was observed in any model. The ORs (95% CI) were 0.84 (0.46–1.53) in the crude model and 0.89 (0.45–1.76) in the fully adjusted model. The ORs (95% CI) for males and females were 0.70 (0.28–1.75) and 1.38 (0.49–4.36), respectively (Supplementary Material Table S5). Similarly, the significant association was not found in the dominant model.

### 3.3. The Interaction of the TFRC rs9846149 Polymorphism and Iron Intake with Gastric Cancer Risk

In our study, we investigated whether there was an effect modification of the association between dietary iron intake and gastric cancer risk by *TFRC* genetic polymorphism. We used the recessive model and dominant model of the rs9846149 genetic polymorphism to observe the interaction.

With regard to recessive model, subjects with a major C allele and higher total iron intake had a significantly lower GC risk than those who had a lower intake (OR = 0.57, 95% CI = 0.41–0.79). In the fully adjusted model, the significant association remained constant (OR = 0.60, 95% CI = 0.41–0.88). However, no protective effect at any level of total iron was observed for males or females. In addition, among individuals in the minor allele group, the relationship of iron with GC risk was null (OR = 1.31, 95% CI = 0.41–4.20). However, we found a significant interaction between the *TFRC* rs9846149 polymorphism and total iron (*p*_interaction_ = 0.035) (Table 4). The interaction appeared to be limited to total iron, and we failed to find an interaction for nonheme iron and heme iron. In contrast, the interaction was not observed in the dominant model. 

## 4. Discussion

Our findings showed the protective effects of total dietary iron, especially nonheme iron, against GC risk. Additionally, a negative relationship of total dietary iron with GC risk was recorded for *H. pylori*-positive subjects, ever-smokers, regular exercisers, and participants without FDFHGC. Nonheme iron exhibited a similar pattern in groups stratified by smoking status, regular exercise, and FDFHGC. Individuals with a major allele of *TFRC* rs9846149 (CC/GC) and higher intake of total iron exhibited significantly lower GC risk than those with a lower intake of iron and a major allele.

To date, many studies have explored dietary iron in relation to GC risk. However, the findings have not been consistent, and studies have focused more on heme iron rather than nonheme iron. For example, the results from a European prospective study that included 444 incident cases reported that GC risk in the participants with the highest consumption of heme iron was higher than that in those with the lowest consumption [HR = 1.67 (1.20–2.34)] [9]. This result was supported by conclusions drawn by other studies [4,19,30]. In contrast, statistical significance was not reached in the Iowa Women’s Health Study and other studies [21,22,23,24].

To our knowledge, the relationships of subtypes of iron with the risk of GC have not been explored thus far. Our study strongly indicated that a significant reduction in GC risk was identified in individuals with higher dietary iron intake. This finding is in agreement with previously reported results [31,32]. In addition, an animal model showed that low dietary iron may result in iron deficiency anemia, enhanced *H. pylori* virulency and increased GC risk [20]. Moreover, another study indicated that body iron status was inversely associated with risk of GC [33]. The protective effect of iron may be linked to its participation in hemoglobin, myoglobin synthesis and the formation of enzymes that are involved in detoxifying free radicals as well as the prevention of oxidative damage. However, high heme iron intake has been demonstrated to have detrimental effects, including cytotoxicity and increase in the formation of endogenous N-nitroso compounds [34]. Additionally, heme iron may be a contributor to free radical formation [9]. The evidence related to carcinogenic effects of heme iron has been reinforced by conclusions from recent studies [4,9]. However, this association did not reach significance in other studies [21,22,23,24]. Importantly, the main food in our population was vegetables, and less consumption of processed meat and red meat was observed [35,36]; as a consequence, the amount of heme iron intake was below the value suggested by the public health recommendations provided by the World Cancer Research Fund [37]. Furthermore, a higher iron consumption from vegetables does not produce a significant amount of carcinogenic nitrogen compounds [38]. Taken together, nonheme iron, but not heme iron, is a major determinant of the link between iron intake and GC risk; thus, a protective effect was observed.

Iron is considered to be necessary for *H. pylori* growth [39,40]. However, the protective effect of iron against GC was recorded for participants with positive *H. pylori* status, who exercise regularly, who smoke cigarettes and who do not have FDFHGC. *H. pylori* infection and cigarette smoking can cause oxidative stress, which can result in DNA damage, and effects on p53, cell proliferation, neoplasm invasion, metastasis and the immune system in the gastrointestinal mucosa [41,42,43]. The literature has documented that nonheme iron is mainly obtained from fruits and vegetables. A hypothesis is that these foods are rich in antioxidants, which have anticancer properties that could counteract these impacts by modulating the methylation of DNA, protecting from and repairing the damage of DNA, inducing detoxification of phase II enzymes and promoting apoptosis [44]. Our finding is consistent with the results of a previous studies [44,45,46].

Iron-regulated genes may influence the risk of cancer and clinical outcome. Several iron metabolism-related genes have been investigated [10,47], but a clear association with *TFRC* is still debated. Accumulating evidence has revealed the participation of *TFRC* in tumor onset and progression, and its overexpression has been proven in many cancers [48]. The expression of *TFRC* is a complicated process due to the participation of regulatory genes such as *IRP1*, *IRP2*, *C-Jun*, *C-Myc*, *cyclin D*, *CREBBP* and *EP300*. The process also involves oxidative stress, inflammation and hypoxia [48]. *TFRC* expression is known to have an impact on the proliferation, migration and invasion of tumor cells via the JAK/STAT pathway, as well as metastasis. Additionally, it is considered a contributor to mitochondrial respiration and reactive oxygen species production and increases the sensitivity of cells to oxidative stress [49]. We hypothesized that transferrin is a necessary component for cell growth and iron-related metabolic processes. *H. pylori* is a common bacterium existing in the stomach of humans, and iron uptake-related genes participate in its colonization and adaptation [15]. *H. pylori*-associated gastritis is well recognized for intestinal type [28]. An insufficient sample size of intestinal type may be responsible for the nonsignificant association between *TFRC* and GC in this study. Additionally, *TFRC* rs9846149 has not been investigated in relation to GC risk [17], and only 5% of participants were GG homozygous. Thus, further studies with a larger sample size are necessary to evaluate this association.

Importantly, we suggest an interaction between *TFRC* rs9846149 and dietary iron in GC progression. The protective effect of iron against GC risk depends on an individual’s genetic background. In detail, our study emphasized that iron has greater effect in individuals with a major allele of *TFRC* rs9846149. The reduction in iron levels in foveolar cells was demonstrated to be linked to *H. pylori*-positive tissues and the overexpression of *TFRC* in these cells. Iron is a vital factor for *H. pylori* growth; therefore, if free iron is limited, iron capture by *H. pylori* through the cells in the gastric mucosa will occur. As a consequence, iron deficiency facilitates serious pathogenic processes caused by *H. pylori*, including carcinogen-induced cancer [15]. The receptor for *H. pylori* adhesins, an iron supplier for these bacteria are key roles of *TFRC* in the process [50]. Thus, inflammation and GC can occur due to iron shortages [15]. This mechanism supports our finding that the protective effect was observed in individuals with a major allele of *TFRC* rs9846149 (CC/GC) and a higher intake of total iron. An additional mechanism should be considered for the interaction between *TFRC* and iron: mammalian target of rapamycin (mTOR) modulates iron homeostasis through variations of cellular iron flux and regulation of *TFR1* stability. Tristetraprolin, an anti-inflammatory protein, was identified as the downstream target of mTOR-bound *TFR1* mRNA and contributed to facilitating its degradation. Therefore, carcinogenesis is induced by *TFR1* due to the regulation of metabolism, inflammation and iron [48].

This study is the first to explore the association and interaction of the *TFRC* gene with the risk of GC. Not only heme iron but also nonheme iron was explored in our study, which allows for the assessment of total iron consumption. To our knowledge, this association has not yet been fully investigated in Korea. In addition, we used a validated SQFFQ. We have sufficient information regarding possible confounding variables, especially *H. pylori* infection and smoking status, which are strong risk factors for GC. However, there are some limitations in our study. We did not focus on other genes that participate in iron metabolism. However, polymorphisms of these genes are not common in the studied population. Similar to any case–control study, the existence of selection bias and recall bias may occur, although great efforts have been made to minimize these biases. The potential relationships in cancer subtypes were not assessed due to insufficient cases. However, non-cardia GC accounted for a high proportion in our study, which is a trend in Asia [3]. Iron supplement intake data was not available to consider as a possible confounding variable in the multivariate analysis.

In conclusion, the study showed the protective effects of total dietary iron, especially nonheme iron, against GC risk, and this association can be modified by *TFRC* rs9846149. However, no epidemiological association was found for this polymorphism and GC risk. This study may contribute evidence to support the recommendation regarding cancer prevention and provide evidence regarding gene-environment interactions. However, it is necessary to clarify the identified association in further studies.

## Figures and Tables

**Table 1:** Distribution of the subjects according to the selected variables.

	Total (*n* = 1128)			Males (*n* = 740)			Females (*n* = 388)		
	Controls (*n* = 754)	Cases (*n* = 374)	*p* ^a^	Controls (*n* = 496)	Cases (*n* = 244)	*p* ^a^	Controls (*n* = 258)	Cases (*n* = 130)	*p* ^a^
**Age (year) ^b^**	53.8 ± 8.9	53.8 ± 9.3	0.967	54.9 ± 8.4	55.0 ± 8.6	0.846	51.9 ± 9.6	51.6 ± 10.2	0.802
**Sex**									
Male	496 (65.8)	244 (65.2)	0.857						
Female	258 (34.2)	130 (34.8)							
**BMI (kg/m^2^)**	24.02 ± 2.9	23.8 ± 3.1	0.375	24.5 ± 2.7	24.2 ± 3.0	0.293	23.1 ± 3.2	23.1 ± 3.0	0.977
<23	275 (36.5)	147 (39.3)	0.631	140 (28.2)	84 (34.4)	0.184	135 (52.3)	63 (48.5)	0.771
23–25	230 (30.5)	106 (28.3)		160 (32.3)	67 (27.5)		70 (27.1)	39 (30.0)	
≥25	248 (32.9)	120 (32.1)		196 (39.5)	93 (38.1)		52 (20.2)	27 (20.7)	
Missing	1 (0.1)	1 (0.3)		0 (0)	0 (0)		1 (0.4)	1 (0.8)	
***H. pylori* infection, *n* (%)**									
Positive	464 (61.5)	346 (92.5)	<0.001	322 (64.9)	228 (93.4)	<0.001	142 (55.0)	118 (90.8)	<0.001
Negative	290 (38.5)	28 (7.5)		174 (35.1)	16 (6.6)		116 (45.0)	12 (9.2)	
Missing	0 (0)	0 (0)		0 (0)	(0)		(0)	(0)	
**First-degree family history of GC, *n* (%)**									
Yes	95 (12.6)	77 (20.6)	<0.001	71 (14.3)	55 (22.5)	0.005	24 (9.3)	22 (16.9)	0.028
No	657 (87.1)	296 (79.2)		423 (85.3)	188 (77.1)		234 (90.7)	108 (83.1)	
Missing	2 (0.3)	1 (0.2)		2 (0.4)	1 (0.4)		0 (0)	0 (0)	
**Smoking status, *n* (%)**									
Current-smoker	153 (20.3)	116 (31.0)	<0.001	149 (30.0)	109 (44.7)	<0.001	4 (1.6)	7 (5.4)	0.037
Ex-smoker	258 (34.2)	109 (29.1)		251 (50.6)	102 (41.8)		7 (2.7)	7 (5.4)	
Non-smoker	343 (45.5)	149 (39.9)		96 (19.4)	33 (13.5)		247 (95.7)	116 (89.2)	
Missing	0 (0)	0 (0)		0 (0)	0 (0)		0 (0)	0 (0)	
**Alcohol intake, *n* (%)**									
Current-drinker	484 (64.2)	226 (60.4)	0.373	369 (74.4)	172 (70.5)	0.393	115 (44.6)	54 (41.5)	0.844
Ex-drinker	58 (7.7)	36 (9.6)		46 (9.3)	30 (12.3)		12 (4.7)	6 (4.6)	
Non-drinker	212 (28.1)	112 (30.0)		81 (16.3)	42 (17.2)		131 (50.7)	70 (53.9)	
Missing	0 (0)	0 (0)		0 (0)	0 (0)		0 (0)	0 (0)	
**Regular exercise, *n* (%)**									
Yes	424 (56.2)	135 (36.1)	<0.001	279 (56.3)	99 (40.6)	<0.001	145 (56.2)	36 (27.7)	<0.001
No	327 (43.4)	239 (63.9)		214 (43.1)	145 (59.4)		113 (43.8)	94 (72.3)	
Missing	3 (0.4)	0 (0)		3 (0.6)	0 (0)		0 (0)	0 (0)	
**Education, *n* (%)**									
Lower high school	109 (14.5)	126 (33.7)	<0.001	64 (12.9)	81 (33.2)	<0.001	45 (17.4)	45 (34.6)	<0.001
High school	225 (29.8)	160 (42.8)		124 (25.0)	104 (42.6)		101 (39.1)	56 (43.1)	
Upper high school	390 (51.7)	87 (23.3)		280 (56.5)	58 (23.8)		110 (42.0)	29 (22.3)	
Missing	30 (4.0)	1 (0.2)		28 (5.6)	1 (0.4)		2 (0.5)	0 (0)	
**Occupation, *n* (%)**									
Professional administrative	144 (19.1)	65 (17.4)	<0.001	108 (21.8)	54 (22.1)	0.007	36 (14.0)	11 (8.5)	0.008
Office, Sales, service	239 (31.7)	107 (28.6)		185 (37.3)	72 (29.5)		54 (20.9)	35 (26.9)	
Laborer, agricultural	117 (15.5)	96 (25.7)		100 (20.2)	76 (31.1)		17 (6.6)	20 (15.4)	
Others, unemployed	251 (33.3)	105 (28.1)		100 (20.1)	41 (16.8)		151 (58.5)	64 (49.2)	
Missing	3 (0.4)	1 (0.2)		3 (0.6)	1 (0.5)		0 (0)	0 (0)	
**Marital status, *n* (%)**									
Married	650 (86.2)	325 (86.9)	0.707	440 (88.7)	219 (89.8)	0.610	210 (81.4)	106 (81.5)	0.973
Others	103 (13.7)	48 (12.8)		55 (11.1)	24 (9.8)		48 (18.6)	24 (18.5)	
Missing	1 (0.1)	1 (0.3)		1 (0.2)	1 (0.4)		0 (0)	0 (0)	
**Monthly income, *n* (%) (10,000 Korean won/mo)**									
<200	132 (17.5)	119 (31.8)	<0.001	74 (14.9)	77 (31.6)	<0.001	58 (22.5)	42 (32.3)	0.046
200–400	311 (41.2)	132 (35.3)		216 (43.5)	94 (38.5)		95 (36.8)	38 (29.2)	
≥400	247 (32.8)	86 (23.0)		153 (30.8)	49 (20.1)		94 (36.4)	37 (28.5)	
Missing	64 (8.5)	37 (9.9)		53 (10.8)	24 (9.8)		11 (4.3)	13 (1.0)	
**Histological subtype of gastric cancer, *n* (%)**									
Intestinal	-	145 (38.8)		-	119 (48.8)		-	26 (20.0)	
Diffuse	-	147 (39.3)		-	69 (28.3)		-	78 (60.0)	
Mixed	-	50 (13.4)		-	36 (14.8)		-	14 (10.8)	
Indeterminate	-	4 (1.1)		-	3 (1.1)		-	1 (0.8)	
Missing		28 (7.4)			17 (7.0)			11 (8.4)	

^a^ Chi-square test and *t*-test were used for categorical variables and continuous variables, respectively; ^b^ the values are presented as the mean ± SD. The association between dietary iron intake and the risk of gastric cancer.

**Table 2:** Difference in the consumption of total energy and iron intakes.

	Total (*n* = 1128)			Males (*n* = 740)			Female (*n* = 388)		
	Controls (*n* = 754)	Cases (*n* = 374)	*p* ^b^	Controls (*n* = 496)	Cases (*n* = 244)	*p* ^b^	Controls (*n* = 258)	Cases (*n* = 130)	*p* ^b^
**Total energy intake (Kcal/day)**	1718.1 ± 546.8	1925.2 ± 613.4	<0.001	1765.1 ± 542.8	2032.9 ± 638.1	<0.001	1627.7 ± 544.1	1723.2 ± 507.8	0.096
**Total iron ^a^ (mg/day)**	13.97 ± 3.8	13.32 ± 3.6	0.005	13.48 ± 3.3	13.03 ± 3.2	0.080	14.92 ± 4.3	13.86 ± 4.1	0.021
**Nonheme iron ^a^ (mg/day)**	10.56 ± 3.0	10.06 ± 3.0	0.008	10.13 ± 2.6	9.76 ± 2.6	0.065	11.38 ± 3.6	10.62 ± 3.5	0.047
**Heme iron ^a^ (mg/day)**	3.44 ± 1.7	3.26 ± 1.5	0.073	3.37 ± 1.6	3.27 ± 1.6	0.373	3.58 ± 1.8	3.26 ± 1.4	0.053

^a^ The iron adjustment for total energy intake was performed using the residuals method; ^b^ Student’s *t*-test was used to calculate *p*-values.

**Table 3:** Association of total iron intake, nonheme iron intake, and heme iron intake with risk of gastric cancer.

Iron (mg/Day)	No. of Controls (%)	No. of Cases (%)	Model I [OR (95% CI)]	Model II [OR (95% CI)]	Model III [OR (95% CI)]
**Total iron ^a^**					
***All***					
T1 (<12.04)	251 (33.3)	147 (39.3)	1	1	1
T2 (12.04–14.76)	250 (33.2)	138 (36.9)	0.94 (0.71–1.26)	1.05 (0.76–1.45)	1.19 (0.84–1.67)
T3 (≥14.76)	253 (33.5)	89 (23.8)	0.60 (0.44–0.82)	0.64 (0.44–0.92)	0.65 (0.45–0.94)
*p* for trend			0.001	0.006	0.018
***Males***					
T1 (<11.67)	166 (33.5)	85 (34.8)	1	1	1
T2 (11.67–14.30)	164 (33.1)	100 (41.0)	1.19 (0.83–1.71)	1.63 (1.06–2.51)	1.71 (1.09–2.69)
T3 (≥14.30)	166 (33.4)	59 (24.2)	0.69 (0.47–1.03)	0.74 (0.47–1.19)	0.81 (0.39–1.31)
*p* for trend			0.053	0.134	0.261
***Females***					
T1 (<12.68)	85 (33.0)	63 (48.5)	1	1	1
T2 (12.68–15.80)	87 (33.7)	35 (26.9)	0.54 (0.33–0.90)	0.66 (0.38–1.17)	0.84 (0.46–1.55)
T3 (≥15.80)	86 (33.3)	32 (24.6)	0.50 (0.30–0.85)	0.60 (0.34–1.08)	0.61 (0.33–1.13)
*p* for trend			0.01	0.089	0.116
**Nonheme iron ^a^**					
***All***					
T1 (<9.10)	251 (33.3)	162 (43.3)	1	1	1
T2 (9.10–11.02)	250 (33.2)	115 (30.8)	0.71 (0.53–0.96)	0.70 (0.50–0.97)	0.73 (0.52–1.04)
T3 (≥11.02)	253 (33.5)	97 (25.9)	0.59 (0.44–0.81)	0.58 (0.41–0.82)	0.64 (0.44–0.92)
*p* for trend			0.001	0.003	0.018
***Males***					
T1 (<8.86)	166 (33.5)	99 (40.6)	1	1	1
T2 (8.86–10.73)	165 (33.3)	79 (32.4)	0.80 (0.56–1.16)	0.79 (0.51–1.21)	0.78 (0.49–1.22)
T3 (≥10.73)	165 (33.2)	66 (27.0)	0.67 (0.46–0.98)	0.63 (0.40–1.00)	0.69 (0.43–1.11)
*p* for trend			0.040	0.052	0.129
***Females***					
T1 (<9.73)	85 (33.0)	66 (50.8)	1	1	1
T2 (9.73–11.69)	86 (33.3)	24 (18.5)	0.36 (0.21–0.63)	0.35 (0.19–0.66)	0.45 (0.23–0.87)
T3 (≥11.69)	87 (33.7)	40 (30.7)	0.59 (0.36–0.97)	0.69 (0.39–1.23)	0.74 (0.40–1.36)
*p* for trend			0.067	0.299	0.408
**Heme iron ^a^**					
***All***					
T1 (<2.60)	251 (33.3)	143 (38.2)	1	1	1
T2 (2.60–3.88)	253 (33.6)	133 (35.6)	0.92 (0.69–1.24)	1.02 (0.73–1.42)	1.06 (0.75–1.51)
T3 (≥3.88)	250 (33.1)	98 (26.2)	0.69 (0.50–0.94)	0.83 (0.58–1.18)	0.81 (0.56–1.17)
*p* for trend			0.017	0.294	0.245
***Males***					
T1 (<2.54)	165 (33.3)	90 (36.9)	1	1	1
T2 (2.54–3.80)	167 (33.7)	87 (35.7)	0.96 (0.66–1.38)	1.11 (0.72–1.69)	1.11 (0.71–1.75)
T3 (≥3.80)	164 (33.0)	67 (27.4)	0.75 (0.51–1.10)	0.90 (0.57–1.40)	0.90 (0.56–1.44)
*p* for trend			0.132	0.599	0.613
***Females***					
T1 (<2.72)	86 (33.3)	54 (41.5)	1	1	1
T2 (2.72–3.99)	87 (33.7)	40 (30.8)	0.73 (0.44–1.21)	0.77 (0.44–1.36)	0.92 (0.50–1.71)
T3 (≥3.99)	85 (33.0)	36 (27.7)	0.68 (0.40–1.13)	0.82 (0.46–1.47)	0.78 (0.42–1.44)
*p* for trend			0.138	0.519	0.417

^a^ The values are presented as tertiles of iron intake. OR: odds ratio, CI: confidence interval. Model I: crude model; Model II: adjusted for age, BMI, first degree family history of GC, smoking status, alcohol consumption, education, occupation, monthly income; Model II: additionally adjusted for Helicobacter pylori infection. In the overall subjects, models II and III were additionally adjusted for gender.

**Table 4:** Associations and interactions of *TFRC* rs9846149 polymorphism (recessive model) and total iron intake with risk of gastric cancer.

**rs9846149**		**CC/GC**			**GG**		***p* Interaction**
**Total**	**Low** **(<12.04)**	**Moderate** **(12.04–14.76)**	**High** **(≥14.76)**	**Low** **(12.04)**	**Moderate** **(12.04–14.76)**	**High** **(≥14.76)**	
No. of controls/cases	240/144	233/131	243/83	11/3	17/7	10/6	
Model I [OR (95% CI)]	1	0.94 (0.70–1.26	0.57 (0.41–0.79)	0.46 (0.13–1.66)	0.69 (0.28–1.70)	1.00 (0.36–2.81)	0.108
Model II [OR (95% CI)]	1	1.04 (0.75–1.45)	0.58 (0.40–0.83)	0.35 (0.08–1.50)	0.75 (0.28–2.00)	1.33 (0.44–3.99)	0.044
Model III [OR (95% CI)]	1	1.15 (0.81–1.62)	0.60 (0.41–0.88)	0.27 (0.06–1.17)	1.07 (0.37–3.07)	1.31 (0.41–4.20)	0.035 *
**Males**	**Low** **(<11.67)**	**Moderate** **(11.67–14.30)**	**High** **(≥14.30)**	**Low** **(<11.67)**	**Moderate** **(11.67–14.30)**	**High** **(≥14.30)**	
No. of controls/cases	157/83	153/95	161/57	9/2	11/5	5/2	
Model I [OR (95% CI)]	1	1.18 (0.81–1.70)	0.67 (0.45–1.00)	0.42 (0.09–1.99)	0.86 (0.29–2.56)	0.76 (0.14–3.98)	0.368
Model II [OR (95% CI)]	1	1.60 (1.05–2.44)	0.74 (0.47–1.17)	0.29 (0.05–1.62)	1.11 (0.33–3.78)	1.49 (0.26–8.49)	0.128
Model III [OR (95% CI)]	1	1.69 (1.08–2.64)	0.79 (0.49–1.29)	0.21 (0.04–1.25)	1.34 (0.36–5.04)	1.77 (0.28–11.34)	0.072
**Females**	**Low** **(<12.68)**	**Moderate** **(12.68–15.80)**	**High** **(≥15.80)**	**Low** **(<12.68)**	**Moderate** **(12.68–15.80)**	**High** **(≥15.80)**	
No. of controls/cases	81/60	81/34	83/29	4/3	6/1	3/3	
Model I [OR (95% CI)]	1	0.57 (0.34–0.95)	0.47 (0.28–0.81)	1.01 (0.22–4.69)	0.23 (0.03–1.92)	1.35 (0.26–6.92)	0.396
Model II [OR (95% CI)]	1	0.64 (0.37–1.13)	0.49 (0.27–0.89)	1.20 (0.23–6.19)	0.26 (0.03–2.53)	2.26 (0.35–14.44)	0.328
Model III [OR (95% CI)]	1	0.82 (0.44–1.52)	0.51 (0.27–1.00)	1.03 (0.18–5.73)	0.31 (0.03–3.24)	3.95 (0.56–28.02)	0.158

C: major allele; G: minor allele. CC/GC: dominant phenotypes; GG: recessive phenotypes. OR: odds ratio; CI: confidence interval. * *p* < 0.05. Model I: crude model; Model II: adjusted for age, BMI, first degree family history of GC, smoking status, alcohol consumption, education, occupation, monthly income; Model II: additionally adjusted for Helicobacter pylori infection. In the overall subjects, models II, and III were additionally adjusted for gender.

## Supplementary Materials

The following are available online at https://www.mdpi.com/article/10.3390/nu13082600/s1, Table S1: Association of total iron intake and nonheme iron intake with risk of gastric cancer stratified by H. pylori infection status, Table S2: Association of total iron intake and nonheme iron intake with risk of gastric cancer stratified by smoking status, Table S3: Association of total iron intake and nonheme iron intake with risk of gastric cancer stratified by regularly exercise, Table S4: Association of total iron intake and nonheme iron intake with risk of gastric cancer stratified by first degree family history of GC, Table S5: Associations of TFRC rs9846149 polymorphism with risk of gastric cancer.
