# Supplementary material for: The Associations of Dietary Iron Intake and the Transferrin Receptor (TFRC) rs9846149 Polymorphism with the Risk of Gastric Cancer: A Case–Control Study Conducted in Korea
Source: Nutrients. 2021 Jul 28;13(8):2600. doi: 10.3390/nu13082600 (PMC8398449; doi:10.3390/nu13082600)
Supplement: Supplementary file 1 [file nutrients-13-02600-s001.zip › nutrients-1304508-supplementary.pdf]

Supplementary material Table S1. Association of total iron intake and nonheme iron intake with risk of gastric cancer stratified by *H. pylori* infection status

	H. pylori-positive				*H. pylori*-negative			
**Iron (mg/day)**	**No. of controls (%)**	**No. of cases (%)**	**Model I [OR (95% CI)]**	**Model II [OR (95% CI)]**	**No. of controls (%)**	**No. of cases (%)**	**Model I [OR (95% CI)]**	**Model II [OR (95% CI)]**
**Total iron[a]**								
All								
T1 (<12.04)	170 (36.6)	138 (39.9)	1	1	81 (27.9)	9 (32.1)	1	1
T2 (12.04–14.76)	142 (30.6)	125 (36.1)	1.08 (0.78–1.51)	1.12 (0.77–1.63)	108 (37.2)	13 (46.4)	1.08 (0.44–2.66)	1.20 (0.44–3.33)
T3 (≥14.76)	152 (32.8)	83 (24.0)	0.67 (0.47–0.95)	**0.66 (0.44–0.98)**	101 (34.8)	6 (21.4)	0.54 (0.18–1.56)	0.40 (0.11–1.47)
p for trend			0.022	**0.035**			0.217	0.151
Males								
T1(<11.67)	117 (36.3)	80 (35.1)	1	1	49 (28.2)	5 (31.3)	1	1
T2 (11.67–14.30)	100 (31.1)	94 (41.2)	1.38 (0.92 – 2.05)	1.65 (1.02–2.67)	64 (36.8)	6 (37.4)	0.92 (0.27–3.19)	1.26 (0.29–5.44)
T3 ((≥14.30)	105 (32.6)	54 (23.7)	0.75 (0.49 – 1.16)	0.81 (0.48–1.36)	61 (35.0)	5 (31.3)	0.80 (0.22–2.93)	0.70 (0.13–3.74)
p for trend			0.165	0.308			0.738	0.678
Females								
T1 (<12.68)	53 (37.3)	58 (49.2)	1	1	32 (27.6)	5 (41.7)	1	1
T2 (12.68–15.80)	41 (28.9)	30 (25.4)	0.67 (0.37 – 1.22)	0.86 (0.44–1.69)	46 (39.7)	5 (41.7)	0.70 (0.19–2.60)	0.53 (0.10–2.80)
T3 (≥15.80)	48 (33.8)	30 (25.4)	0.57 (0.32 – 1.03)	0.65 (0.34–1.26)	38 (32.7)	2 (16.6)	0.34 (0.06–1.86)	0.47 (0.05–4.29)
p for trend			0.063	0.202			0.206	0.451
**Nonheme iron[a]**								
All								
T1 (<9.10)	170 (36.6)	150 (43.4)	1	1	81 (27.9)	12 (42.9)	1	1
T2 (9.10–11.02)	153 (33.0)	106 (30.6)	0.79 (0.56–1.09)	0.77 (0.53–1.13)	97 (33.5)	9 (32.1)	0.63 (0.25–1.56)	0.58 (0.20–1.68)
T3 (≥11.02)	141 (30.4)	90 (26.0)	0.72 (0.51–1.02)	0.70 (0.47–1.05)	112 (38.6)	7 (25.0)	0.42 (0.16–1.12)	0.28 (0.09–1.94)
p for trend			0.067	0.093			0.088	0.039
Males								
T1 (<8.86)	112 (34.8)	95 (41.7)	1	1	54 (31.0)	4 (25.0)	1	1
T2 (8.86–10.73)	109 (33.9)	73 (32.0)	0.79 (0.53–1.18)	0.70 (0.43–1.13)	56 (32.2)	6 (37.5)	1.45 (0.39–5.41)	1.26 (0.26–6.12)

T3 (≥10.73)	101 (31.4)	60 (26.3)	0.70 (0.46–1.07)	0.67 (0.40–1.12)	64 (36.8)	6 (37.5)	1.27 (0.34–4.72)	1.08 (0.22–5.34)
p for trend			0.099	0.136			0.789	0.948
Females								
T1 (<9.73)	56 (39.4)	59 (50.0)	1	1	29 (25.0)	7 (58.3)	1	1
T2 (9.73–11.69)	41 (28.9)	21 (17.8)	0.49 (0.26–0.92)	0.49 (0.24–1.03)	45 (38.8)	3 (25.0)	0.28 (0.07–1.16)	0.25 (0.04–1.51)
T3 (≥11.69)	45 (31.7)	38 (32.2)	0.80 (0.46–1.41)	0.89 (0.46–1.71)	42(36.2)	2 (16.7)	1.20 (0.04–1.12)	0.26 (0.03–2.05)
p for trend			0.511	0.830			0.057	0.176

[a] *The values are presented as tertiles of iron intake.*

OR: odds ratio, CI: confidence interval

***Model I**: crude model; **Model II**: adjusted for age, first degree family history of GC, BMI, alcohol consumption, smoking status, education, occupation, monthly income. In the overall subjects, model II was additionally adjusted for gender.*

Supplementary material Table S2. Association of total iron intake and nonheme iron intake with risk of gastric cancer stratified by smoking status

	Ever-smoker (current-smoker and ex-smoker)				Non-smoker			
**Iron (mg/day)**	**No. of controls (%)**	**No. of cases (%)**	**Model I [OR (95% CI)]**	**Model II [OR (95% CI)]**	**No. of controls (%)**	**No. of cases (%)**	**Model I [OR (95% CI)]**	**Model II [OR (95% CI)]**
**Total iron[a]**								
All								
T1 (<12.04)	152 (37.0)	90 (40.0)	1	1	99 (28.9)	57 (38.3)	1	1
T2 (12.04–14.76)	137 (33.3)	94 (41.8)	1.16 (0.80–1.68)	1.37 (0.86–2.18)	113 (32.9)	44 (29.5)	0.68 (0.42–1.09)	0.95 (0.54–1.67)
T3 (≥14.76)	122 (29.7)	41 (18.2)	0.57 (0.37– 0.88)	**0.51 (0.29–0.88)**	131 (38.2)	48 (32.2)	0.64 (0.40–1.01)	0.76 (0.44–1.32)
p for trend			0.012	**0.016**			0.075	0.223
Males								
T1(<11.67)	131 (32.8)	76 (36.0)	1	1	35 (36.5)	9 (27.3)	1	1
T2 (11.67–14.30)	133 (33.3)	85 (40.3)	1.10 (0.74–1.63)	1.35 (0.83–2.21)	31 (32.3)	15 (45.4)	1.88 (0.72–4.90)	18.81 (2.93–121.0)
T3 ((≥14.30)	136 (34.0)	50 (23.7)	0.63 (0.41–0.97)	0.59 (0.35–1.01)	30 (31.3)	9 (27.3)	1.17 (0.41–3.32)	3.52 (0.64–121.7)
p for trend			0.029	0.035			0.870	0.529
Females								
T1 (<12.68)	4 (33.7)	8 (57.1)	1	1	81 (32.8)	55 (47.4)	1	1
T2 (12.68–15.80)	4 (33.7)	4 (28.6)	0.50 (0.08–3.13)	-	83 (33.6)	31 (26.7)	0.55 (0.32–0.94)	0.79 (0.42–1.48)
T3 (≥15.80)	3 (27.3)	2 (14.3)	0.33 (0.04–2.88)	-	83 (33.6)	30 (25.9)	0.53 (0.31–0.91)	0.59 (0.31–1.10)
P for trend			0.299	-			0.024	0.100
**Nonheme iron[a]**								
All								
T1 (<9.10)	154 (37.5)	107 (47.6)	1	1	97 (28.3)	55 (36.9)	1	1
T2 (9.10–11.02)	140 (34.1)	70 (31.1)	0.72 (0.49–1.05)	0.62 (0.39–1.00)	110 (32.1)	45 (30.2)	0.72 (0.45–1.17)	0.91 (0.50–1.60)
T3 (≥11.02)	117 (28.4)	48 (21.3)	0.59 (0.30–0.90)	**0.54 (0.32–0.91)**	136 (39.7)	49 (32.9)	0.64 (0.40–1.01)	0.76 (0.43–1.33)
p for trend			0.012	**0.018**			0.068	0.215
Males								
T1 (<8.86)	133 (33.3)	87 (41.2)	1	1	33 (34.4)	12 (36.4)	1	1
T2 (8.86–10.73)	134 (33.4)	69 (32.7)	0.79 (0.53–1.17)	0.69 (0.42–1.13)	31 (32.3)	10 (30.3)	0.89 (0.34–2.35)	1.79 (0.45-7.05)

T3 (≥10.73)	133 (33.3)	55 (26.1)	0.63 (0.42–0.96)	**0.55 (0.33–0.93)**	32(33.3)	11 (33.3)	0.95 (0.37–2.45)	1.20 (0.32–4.59)
p for trend			0.031	**0.028**			0.923	0.880
Females								
T1 (<9.73)	4 (36.4)	11 (78.6)	1	1	81 (32.8)	55 (47.4)	1	1
T2 (9.73–11.69)	6 (54.6)	1 (7.1)	0.06 (0.01–0.67)	-	80 (32.4)	23 (19.8)	0.42 (0.24–0.75)	0.51 (0.26–1.00)
T3 (≥11.69)	1 (9.0)	2 (14.3)	0.73 (0.05–10.39)	-	86 (34.8)	38 (32.8)	0.65 (0.39–1.09)	0.76 (0.41–1.42)
p for trend			0.370	-			0.166	0.470

[a] *The values are presented as tertiles of iron intake.*

OR: odds ratio, CI: confidence interval

Model I*: crude model;* ***Model II****: adjusted for age, first degree family history of GC, alcohol consumption, BMI, education, occupation, monthly income, H. pylori infection. In the overall subjects, model II was additionally adjusted for gender*

Supplementary material Table S3. Association of total iron intake and nonheme iron intake with risk of gastric cancer stratified by regularly exercise

	No				Yes			
**Iron (mg/day)**	**No. of controls (%)**	**No. of cases (%)**	**Model I [OR (95% CI)]**	**Model II [OR (95% CI)]**	**No. of controls (%)**	**No. of cases (%)**	**Model I [OR (95% CI)]**	**Model II [OR (95% CI)]**
**Total iron[a]**								
All								
T1 (<12.04)	134 (41.0)	97 (40.6)	1	1	116 (27.4)	50 (37.0)	1	1
T2 (12.04–14.76)	112 (34.3)	89 (37.2)	1.10 (0.75–1.61)	1.33 (0.84–2.11)	136 (32.1)	49 (36.3)	0.84 (0.53–1.33)	0.95 (0.55–1.64)
T3 (≥14.76)	81 (24.8)	53 (22.2)	0.90 (0.59–1.40)	0.82 (0.48–1.37)	172 (40.5)	36 (26.7)	0.49 (0.30–0.79)	**0.47 (0.26–0.84)**
p for trend			0.665	0.456			0.003	**0.008**
Males								
T1(<11.67)	96 (44.9)	52 (35.9)	1	1	69 (24.7)	33 (33.3)	1	1
T2 (11.67–14.30)	67 (31.3)	57 (39.3)	1.57 (0.96–2.56)	2.64 (1.34–5.21)	95 (34.1)	43 (43.4)	0.95 (0.55–1.64)	1.16 (0.60–2.26)
T3 ((≥14.30)	51 (23.8)	36 (24.8)	1.30 (0.76–2.25)	1.53 (0.75–3.15)	115 (41.2)	23 (23.3)	0.42 (0.23–0.70)	**0.41 (0.20–0.85)**
p for trend			0.316	0.305			0.003	**0.009**
Females								
T1 (<12.68)	40 (35.4)	50 (53.2)	1	1	45 (31.0)	13 (36.1)	1	1
T2 (12.68–15.80)	42 (37.2)	22 (23.4)	0.42 (0.22–0.81)	0.57 (0.26–1.26)	45 (31.0)	13 (36.1)	1.00 (0.42–0.39)	1.39 (0.47–4.11)
T3 (≥15.80)	31 (27.4)	22 (23.4)	0.57 (0.29–1.13)	0.57 (0.25–1.28)	55 (37.9)	10 (27.8)	0.63 (0.25–1.57)	0.77 (0.24–2.40)
p for trend			0.089	0.163			0.297	0.584
**Nonheme iron[a]**								
**All**								
T1 (<9.10)	140 (42.8)	108 (45.2)	1	1	109 (25.7)	54 (40.0)	1	1
T2 (9.10–11.02)	102 (31.2)	70 (29.3)	0.89 (0.60–1.32)	0.91 (0.57–1.46)	148 (34.9)	45 (33.3)	0.61 (0.39–0.98)	0.55 (0.31–0.97)
T3 (≥11.02)	85 (26.0)	61 (25.5)	0.93 (0.62-1.41)	0.83 (0.50–1.38)	167 (39.4)	36 (66.7)	0.44 (0.27–0.71)	**0.45 (0.25–0.81)**
p for trend			0.722	0.476			0.001	**0.011**
Males								
T1 (<8.86)	99 (46.3)	58 (40.0)	1	1	65 (23.3)	41 (41.4)	1	1
T2 (8.86–10.73)	66 (30.8)	47 (32.4)	1.22 (0.74–1.99)	1.30 (0.68–2.47)	99 (35.5)	32 (32.3)	0.51 (0.29–0.90)	0.38 (0.19–0.76)

T3 (≥10.73)	49 (22.9)	40 (27.6)	1.39 (0.82–2.36)	1.46 (0.73–2.89)	115 (41.2)	26 (26.3)	0.36 (0.20–0.64)	**0.29 (0.14–0.60)**
p for trend			0.214	0.282			<0.001	**0.002**
Females								
T1 (<9.73)	39 (34.1)	53 (56.4)	1	1	46 (31.7)	13 (36.1)	1	1
T2 (9.73–11.69)	40 (35.4)	16 (17.0)	0.29 (0.14–0.60)	0.37 (0.16–0.87)	46 (31.7)	8 (22.2)	0.62 (0.23–1.63)	0.85 (0.26–2.76)
T3 (≥11.69)	34 (30.1)	25 (26.6)	0.54 (0.28–1.05)	0.54 (0.25–1.21)	53 (36.6)	15 (41.7)	1.00 (0.43–2.32)	1.44 (0.48–4.33)
p for trend			0.081	0.153			0.835	0.464

[a] *The values are presented as tertiles of iron intake.*

OR: odds ratio, CI: confidence interval

***Model I**: crude model; **Model II**: adjusted for age, first degree family history of GC, BMI, alcohol consumption, smoking status, education, occupation, monthly income, H. pylori infection. In the overall subjects, model II was additionally adjusted for gender.*

Supplementary material Table S4. Association of total iron intake and nonheme iron intake with risk of gastric cancer stratified by first degree family history of GC.

	Yes				No			
**Iron (mg/day)**	**No. of controls (%)**	**No. of cases (%)**	**Model I [OR (95% CI)]**	**Model II [OR (95% CI)]**	**No. of controls (%)**	**No. of cases (%)**	**Model I [OR (95% CI)]**	**Model II [OR (95% CI)]**
**Total iron[a]**								
All								
T1 (<12.04)	37 (39.0)	35 (45.5)	1	1	213 (32.4)	112 (37.8)	1	1
T2 (12.04–14.76)	35 (36.8)	25 (32.5)	0.76 (0.38–1.51)	0.97 (0.37–2.54)	214 (32.6)	113 (38.2)	1.00 (0.73–1.39)	1.19 (0.81–1.74)
T3 (≥14.76)	23 (24.2)	17 (22.1)	0.78 (0.36–1.70)	0.82 (0.28–2.41)	230 (35.0)	71 (24.0)	0.59 (0.41–0.83)	**0.60 (0.39–0.91)**
p for trend			0.518	0.728			0.002	**0.011**
Males								
T1(<11.67)	27 (38.0)	25 (45.5)	1	1	138 (32.6)	60 (31.9)	1	1
T2 (11.67–14.30)	26 (36.6)	17 (30.9)	0.71 (0.31–1.60)	1.30 (0.38–4.51)	137 (32.4)	83 (44.2)	1.39 (0.93–2.10)	1.87 (1.13–3.08)
T3 (≥14.30)	18 (25.4)	13 (23.6)	0.78 (0.32–1.91)	0.94 (0.25–3.53)	148 (35.0)	45 (23.9)	0.70 (0.45–1.10)	0.74 (0.43–1.28)
p for trend			0.569	0.933			0.074	0.166
Females								
T1 (<12.68)	12 (50.0)	10 (45.5)	1	1	73 (31.2)	53 (49.1)	1	1
T2 (12.68–15.80)	7 (29.2)	5 (22.7)	0.86 (0.21–3.55)	2.70 (0.24–30.4)	80 (34.2)	30 (27.8)	0.52 (0.30–0.89)	0.74 (0.39–1.42)
T3 (≥15.80)	5 (20.8)	7 (31.8)	1.68 (0.41–6.96)	6.36 (0.45–89.4)	81 (34.6)	25 (23.1)	0.43 (0.24–0.75)	0.51 (0.26–1.00)
p for trend			0.492	0.151			0.004	0.053
**Nonheme Iron[a]**								
Total								
T1 (<9.10)	42 (44.2)	36 (46.8)	1	1	209 (31.8)	126 (42.6)	1	1
T2 (9.10–11.02)	31 (32.6)	26 (33.8)	0.98 (0.49–1.94)	1.06 (0.42–2.72)	218 (33.2)	88 (29.7)	0.67 (0.48–0.93)	0.72 (0.49–1.07)
T3 (≥11.02)	22 (23.2)	15 (19.4)	0.80 (0.36–1.76)	0.90 (0.29–2.82)	230 (35.0)	82 (27.7)	0.59 (0.42–0.83)	**0.61 (0.41–0.91)**
p for trend			0.583	0.874			0.003	**0.019**
Males								
T1 (<8.86)	27 (38.0)	26 (47.3)	1	1	139 (32.9)	73 (38.8)	1	1
T2 (8.86–10.73)	27 (38.0)	18 (32.7)	0.69 (0.31–1.55)	0.73 (0.23–2.32)	137 (32.4)	60 (31.9)	0.83 (0.55–1.26)	0.79 (0.48–1.31)

T3 (≥10.73)	17 (23.9)	11 (20.0)	0.67 (0.27–1.70)	0.82 (0.21–3.31)	147 (34.7)	55 (29.3)	0.71 (0.47–1.08)	0.65 (0.39–1.09)
p for trend			0.375	0.755			0.117	0.107
Females								
T1 (<9.73)	12 (50.0)	8 (36.4)	1	1	73 (31.2)	58 (53.7)	1	1
T2 (9.73–11.69)	8 (33.3)	7 (31.8)	1.31 (0.34–5.08)	1.16 (0.09–14.95)	78 (33.3)	17 (15.7)	0.27 (0.15–0.51)	0.37 (0.18–0.77)
T3 (≥11.69)	4 (16.7)	7 (31.8)	2.63 (0.57–12.0)	14.13 (0.71–280.75)	83 (35.5)	33 (30.6)	0.50 (0.29–0.85)	0.63 (0.33–1.21)
p for trend			0.214	0.082			0.023	0.226

[a] *The values are presented as tertiles of iron intake.*

OR: odds ratio, CI: confidence interval

Model I*: crude model;* ***Model II****: adjusted for age, BMI, alcohol consumption, smoking status, education, occupation, monthly income, H. pylori infection. In the overall subjects, model II was additionally adjusted for gender.*

Supplementary material Table S5. Associations of *TFRC* rs9846149 polymorphism with risk of gastric cancer

rs9846149	Model	Genotype	No (%)		OR (95% CI)		
			Controls	Cases	Model I	Model II	Model III
**Total**	**Recessive**	**CC/GC**	716 (95.0)	358 (95.7)	1	1	1
		**GG**	38 (5.0)	16 (4.3)	0.84 (0.46–1.53)	0.87 (0.46-1.67)	0.89 (0.45–1.76)
**Males**	**Recessive**	**CC/GC**	471 (95.0)	235 (96.3)	1	1	1
		**GG**	25 (5.0)	9 (3.7)	0.72 (0.33–1.57)	0.73 (0.31-1.73)	0.70 (0.28–1.75)
**Females**	**Recessive**	**CC/GC**	245 (95.0)	123 (94.6)	1	1	1
		**GG**	13 (5.0)	7 (5.4)	1.07 (0.42–2.76)	1.35 (0.48-3.77)	1.38 (0.49–4.36)

C: major allele; G: minor allele

CC/GC: dominant phenotypes; GG: recessive phenotypes

OR: odds ratio, CI: confidence interval

***Model I**: crude model; **Model II**: adjusted for age, BMI, first degree family history of GC, smoking status, alcohol consumption, education, occupation, monthly income; **Model II**: additionally adjusted for Helicobacter pylori infection. In the overall subjects, models II, and III were additionally adjusted for gender.*
